# Bedarf an Remote Care in der Cochleaimplantat-Nachsorge

**DOI:** 10.1007/s00106-025-01667-4

**Published:** 2025-09-05

**Authors:** A. Barthel, J. J. -H. Park, A. Breinhild-Olsen, V. Mazur, M. Radüg, S. Bohmann, S. Kreikemeier

**Affiliations:** 1 Hörzentrum Hagen Südwestfalen, Katholisches Krankenhaus Hagen, St.-Josefs-Hospital, Universität Witten/Herdecke, Dreieckstraße 15, 58097 Hagen, Deutschland; 2 Fachbereich Akustik und Audiologie, Hochschule Aalen, Aalen, Deutschland

**Keywords:** Cochleaimplantat-Fernanpassung, Telemedizin, Hörrehabilitation, Digitale Gesundheitsanwendungen, Nachfrageanalyse, Remote cochlear implant fitting, Telemedicine, Auditory rehabilitation, Digital health applications, Demand analysis

## Abstract

**Hintergrund:**

Remote Care (RC) eröffnet neue Möglichkeiten in der Cochleaimplantat(CI)-Nachsorge, wie z. B. Echtzeit-Fernanpassungen, Implantat- und Sprachprozessorüberprüfungen sowie Hörkontrollen mit Hilfe von Smartphone-Apps. Diese Anwendungen bieten CI-Tragenden und Kliniken zahlreiche Potenziale, die erfolgreiche Implementierung in den Alltag hängt jedoch von verschiedenen Faktoren ab. Bisher gibt es wenige Studien, die den Bedarf an RC seitens der CI-Tragenden untersucht haben.

**Ziel der Arbeit:**

Die Studie untersucht herstellerunabhängig den Bedarf an RC in der CI-Nachsorge aus Sicht CI-Tragender. Es wird analysiert, welche Aspekte CI-Tragenden in der Nachsorge wichtig sind, welche Argumente sie für oder gegen die Nutzung von RC anführen und welche RC-Funktionen sie nutzen würden.

**Methodik:**

Die Arbeit basiert auf einer Befragung im Hörzentrum Hagen Südwestfalen im Dezember 2024 mittels eines selbstentwickelten Fragebogens. Insgesamt wurden 86 Antworten ausgewertet.

**Ergebnisse:**

Auf die Frage, ob sie RC nutzen würden, haben 64,3 % der Befragten mit *Ja* oder *Eher ja* geantwortet. Durch eine regelmäßige Smartphone-Nutzung erfüllen 82,6 % die grundsätzliche Voraussetzung für RC. Im Median ist der fehlende persönliche Kontakt das stärkste Argument gegen die Nutzung von RC. Jedoch sieht ein Großteil der Befragten als Vorteil Zeitersparnis.

**Schlussfolgerung:**

Insgesamt zeigt die Mehrheit der Befragten ein Interesse an RC. Dennoch wird der persönliche Kontakt zum Fachpersonal weiterhin als wichtig erachtet. Auch nimmt das Interesse an RC mit zunehmendem Alter signifikant ab (*p* < 0,05). Zukünftig stellt RC eine innovative Ergänzung zu der herkömmlichen CI-Nachsorge mit Vor-Ort-Terminen dar, welche Nachfrage finden wird.

## Abstract (en)

**Background:**

Remote care (RC) opens up new possibilities in cochlea implant (CI) aftercare, such as real-time remote fittings, implant and speech processor checks, and hearing checks using smartphone apps. These applications offer CI recipients and clinics numerous options, but successful implementation in everyday life depends on various factors. To date, there have been few studies that have investigated the demand of RC on the part of CI recipients.

**Objectives:**

The study analyses the demand for RC in CI aftercare from the perspective of CI recipients, regardless of the manufacturer. It demonstrates which aspects are important to the participants in CI aftercare, which arguments they cite for or against the use of RC, and which RC functions they would like to use.

**Methods:**

The paper is based on a survey conducted at Hörzentrum Hagen Südwestfalen in December 2024 using a self-developed questionnaire. A total of 86 answers were evaluated.

**Results:**

A total of 64.3% of respondents answered *yes* or *rather yes* to the question of whether they would use RC. In all, 82.6% fulfill the basic requirement for RC through regular smartphone use. The lack of personal contact is the strongest argument against the use of RC. However, the majority of respondents see an advantage in saving time.

**Conclusion:**

Overall, the majority of respondents showed an interest in RC. Nevertheless, personal contact with specialized staff is still considered important. Interest in RC also decreases significantly with increasing age (*p* < 0.05). In the future, RC will be an innovative addition to conventional CI aftercare with on-site appointments, which will be in demand.

Zunehmend existieren digitale Gesundheitsanwendungen, die Patienten und Patientinnen im Alltag unterstützen und Wege zu spezialisierten Einrichtungen ersparen können. Auch in der Cochleaimplantat(CI)-Versorgung gibt es Apps für z. B. Hörtraining und Sprachprozessor-Checks, welche von CI-Tragenden eigenständig durchgeführt werden können [5, 23]. Des Weiteren sind Sprachprozessor-Einstellungen via Fernversorgung (Remote Care, RC) möglich [1]. RC bietet großes Potenzial, oft ist jedoch persönlicher Kontakt in der CI-Nachsorge ein wichtiger Faktor für eine erfolgreiche Hörrehabilitation.

## Remote Care und CI-Fernanpassungen

Ein essenzieller Bestandteil der Versorgung mit CI ist neben der Operation die Hörrehabilitation und Nachsorge. Nur durch regelmäßige Einstellungen des Sprachprozessors und Rehabilitationsmaßnahmen, wie z. B. Hörtraining, kann eine optimale Hörwahrnehmung und ein bestmögliches Sprachverstehen erreicht werden [6, 23]. Traditionell erfolgt dies in einem klinischen Umfeld oder in Rehabilitationseinrichtungen, zu denen CI-Tragende ambulant oder stationär anreisen [2, 20].

Mit der zunehmenden Nutzung von Telemedizin und RC ergeben sich neue Möglichkeiten in der medizinischen und rehabilitativen Versorgung. „Telemedizin ermöglicht es, unter Einsatz audiovisueller Kommunikationstechnologien trotz räumlicher Trennung z. B. Diagnostik, Konsultation, Monitoring und medizinische Notfalldienste anzubieten“ [3]. Vermehrt stehen auch in der CI-Nachsorge RC-Möglichkeiten zur Verfügung. Darunter werden ferntelemetrische Messungen und Fernanpassungen der Sprachprozessoren verstanden, die mittels Datenfernverbindung und durch Unterstützung von Video- und Tonübertragung stattfinden können [23].

In einem, in Studien häufig untersuchten, Szenario ist eine Person mit CI über das Internet mit einer auf CI-Einstellungen spezialisierten RC-Fachkraft in einer Klinik verbunden. Zur Unterstützung ist meist eine Fachkraft vor Ort anwesend, die jedoch nicht auf die spezifische CI-Anpassung spezialisiert ist. Meist wird die Verbindung zur RC-Fachkraft durch eine internetbasierte Video- und Tonübertragung ergänzt, die bei Bedarf durch eine zusätzliche Telefonverbindung erweitert werden kann [7, 11, 16]. Die Durchführbarkeit, die Sicherheit und die Effektivität von CI-Fernanpassungen sowie die Vergleichbarkeit der Ergebnisse mit Vor-Ort-Anpassungen wurden in verschiedenen Studien belegt [7, 8, 11, 13, 18, 20, 21, 24]. Zudem zeigen sich sowohl CI-Tragende als auch Fachkräfte zufrieden mit dem Verfahren [10, 18, 21]. Die positiven Ergebnisse ließen sich ebenfalls bereits bei der Fernanpassung mit Kindern nachweisen [20]. Ein Großteil der Studienteilnehmenden sprach sich dafür aus, RC auch zukünftig nutzen zu wollen [7, 10]. Vor allem CI-Tragende mit einem weiten Anfahrtsweg zur betreuenden Klinik sehen einen Vorteil in RC [24].

Moderne Technologien ermöglichen mittlerweile eine Echtzeit-Fernanpassung und -überprüfung von CI über Smartphone-Apps. Dadurch können CI-Tragende Anpassungen von zu Hause aus vornehmen lassen [1, 5]. Eine andere Möglichkeit für Kliniken besteht darin, Konfigurationen des Sprachprozessors als Backup in einer Cloud zu speichern [14]. Einige Anwendungen erlauben außerdem die Fernüberprüfung des Gehörs, darunter Ton- und Sprachmessungen sowie technische Prüfungen im Rahmen von Impedanzmessungen oder Sprachprozessor-Überprüfungen. Diese Kontrollen können von den CI-Tragenden z. T. eigenständig durchgeführt werden, und die Ergebnisse werden anschließend an die betreuende Einrichtung übermittelt [1, 5]. In einer Studie zur Untersuchung der Anwendung *Remote Check* (virtuelles Assessment-Tool der Fa. Cochlear Ltd., Sydney, Australien) wurden 94 % der Auffälligkeiten korrekt erkannt, und in 99 % der Fälle entsprach die automatische Einschätzung der Expertenmeinung, ob ein Klinikbesuch erforderlich sei [4, 12]. Darüber hinaus gibt es erste Ansätze für Self-Fitting-Methoden, die auf künstlicher Intelligenz basieren und CI-Tragenden ermöglichen, selbstständig Anpassungen vorzunehmen [15]. Auch die Lebensqualität kann über digitale Fragebögen in Form von RC erfasst werden [16], und es gibt eine Reihe von Computer- oder App-basierten Hörtrainingsanwendungen, zu denen im deutschsprachigen Raum jedoch keine größeren Wirksamkeitsstudien vorliegen [23].

RC eröffnet in der CI-Versorgung demnach zahlreiche Potenziale, wie die Einsparung von Anfahrts- und Wartezeiten sowie die Reduktion von Kosten für CI-Tragende [24]. Zudem können Arbeits- und Schulausfälle minimiert werden [10, 21]. Zukünftig könnte RC bei der Versorgung von CI-Tragenden mit altersbedingter Immobilität eine größere Relevanz entfalten, indem es eine ortsunabhängige Betreuung ermöglicht und so eine durchgängige Versorgung in allen Lebensphasen sicherstellt [9]. Auch implantierende Kliniken und Rehabilitationszentren können von RC profitieren. Angesichts der stetig steigenden Zahl CI-Tragender kann RC dazu beitragen, dem wachsenden Bedarf an Ressourcen gerecht zu werden [11, 16, 21].

RC unterliegt jedoch auch Herausforderungen und Limitationen. Die Methode ist nicht für alle Personen gleichermaßen geeignet. Studien zeigen, dass untersuchte Teilnehmenden-Kollektive häufig nicht repräsentativ für durchschnittliche CI-Tragende sind, da sie gezielt für Studien ausgewählt wurden [20, 21]. RC hat außerdem Grenzen bei CI-Tragenden mit Fazialisnervstimulation, Sehbehinderungen oder kognitiven Einschränkungen [24]. Ebenso profitieren CI-Tragende, die eine intensive Betreuung, umfassende Beratung oder komplexe Fehlerbehebung benötigen, von RC weniger als erfahrene CI-Tragende, die lediglich routinemäßige Kontrollen benötigen [4, 16, 21]. Die praktische Umsetzung von RC erfordert eine stabile Internetverbindung sowie die technische Funktionsfähigkeit aller Geräte und Systeme der Beteiligten [13, 16]. Dies schließt auch die sichere Smartphone-Bedienung durch CI-Tragende mit ein. Ein weiterer Aspekt ist die einheitliche Durchführung von Ton- und Sprachmessungen, welche idealerweise unter standardisierten Bedingungen in einer schallgedämmten Hörkabine erfolgen sollten. In häuslicher Umgebung können signifikante Abweichungen auftreten [8].

Zusammengefasst kann RC bei Personen mit ausreichender Erfahrung im Umgang mit ihrem Sprachprozessor und einer relativen Stabilität im Anpassprozess viele Vorteile bieten [4, 9]. Jedoch bleibt offen, in welchem Umfang und auf welche Weise diese Methode langfristig in den klinischen und rehabilitativen Alltag integriert werden sollte. Insbesondere stellt sich die Frage, ob RC den persönlichen Austausch und den sozialen Kontakt von CI-Tragenden untereinander und zum Fachpersonal ersetzen kann [2, 10, 18]. Der Bedarf und das Interesse an RC in der CI-Versorgung wurden bisher in wenigen Studien untersucht. Eine Umfrage von Cochlear Ltd. zeigt, dass 63 % der befragten Erwachsenen mit Hörverlust und einem Durchschnittsalter von 70 Jahren RC nutzen würden [5]. Die Teilnehmenden einer Studie von Advanced Bionics (Stäfa, Schweiz) haben überwiegend zugestimmt, dass die Fernanpassung der Vor-Ort-Anpassung ähnelt und das gleiche Niveau der Versorgung erreicht werden kann [1]. Eine Studie von Maruthurkkara et al. ergab, dass 77 % der Befragten zufrieden damit wären, wenn die Notwendigkeit von Vor-Ort-Besuchen in der Klinik auf den Ergebnissen der *Remote-Check*-Anwendung basieren würde [12].

Die vorliegende Studie untersucht herstellerunabhängig den Bedarf an RC in der CI-Nachsorge aus Sicht CI-Tragender mit Hilfe eines selbstentwickelten Fragebogens. Analysiert werden wichtige Aspekte in der Nachsorge sowie Argumente für und gegen RC. Darüber hinaus wird geprüft, ob und welche Funktionen im Bereich RC genutzt werden würden. Es wird erwartet, dass ein grundsätzliches Interesse an RC besteht, primär als Ergänzung zu Vor-Ort-Anpassungen. Gleichzeitig wird angenommen, dass viele CI-Tragende weiterhin Wert auf persönlichen Kontakt zum Fachpersonal legen. Außerdem besteht die Annahme, dass das Interesse an RC größer wird, je jünger die Teilnehmenden sind und je weiter der Anfahrtsweg zur betreuenden Einrichtung ist. Die Ergebnisse der Umfrage sollen Hinweise liefern, ob, wie und in welchem Umfang RC als integraler Bestandteil der CI-Nachsorge implementiert werden kann.

## Methodik

Die vorliegende Arbeit basiert auf einer anonymen Befragung von CI-Tragenden mittels eines selbsterstellten Fragebogens. Die Umfrage wurde im Dezember 2024 postalisch und digital an CI-Tragende versendet, welche sich im Hörzentrum Hagen Südwestfalen in der Nachsorge befanden. Teilnehmende konnten den Fragebogen handschriftlich oder online über einen Link bzw. QR-Code über Unipark (Tivian) ausfüllen. Teilnahmeberechtigt waren Personen, die mindestens mit einem CI versorgt waren.

Der vierseitige Fragebogen begann mit einer Einführung in das RC-Konzept und dessen möglicher Anwendung in der CI-Nachsorge, um auch Personen ohne Vorwissen zu RC die Beantwortung der Fragen zu ermöglichen. Der Hauptteil umfasste elf Fragen: Zunächst wurden allgemeine Daten, wie Alter (Frage 1), Geschlecht (Frage 2), Smartphone-Nutzung (Frage 3), Jahr der Implantation (Frage 4), Anfahrtsweg zur betreuenden Klinik (Frage 5) sowie die Kenntnis von RC in der CI-Nachsorge vor der Umfrage (Frage 7), erhoben. Die weiteren Fragen bezogen sich auf die Wichtigkeit spezifischer Aspekte in der CI-Nachsorge (Frage 6), Argumente für und gegen die Nutzung von RC (Fragen 8 und 9) sowie potenzielle Nutzungsabsichten und gewünschte RC-Funktionen (Fragen 10 und 11).

Die Antwortmöglichkeiten der Fragen 6, 8, 9, 10 und 11 wurden als symmetrische Likert-Skalen gestaltet. Zusätzlich konnten die Teilnehmenden bei den Fragen 6, 8, 9 und 10 im Freitextfeld unter *Sonstiges* weitere Aspekte ergänzen. In der digitalen Version wurden die Aspekte bei den Fragen 6, 8, 9 und 10 in randomisierter Reihenfolge angezeigt, in der Papierversion war dies nicht möglich. Der Fragebogen wurde bewusst kurz und einfach gehalten, um eine vollständige und bewusste Beantwortung zu fördern. Zudem wurde auf die freiwillige und anonyme Teilnahme hingewiesen. Personen, die online an der Umfrage teilnahmen, stimmten der Erhebung und Verarbeitung ihrer Daten durch das Setzen eines Häkchens zu. Den postalisch versendeten Umfragen lag ein Anschreiben bei, das darauf hinwies, dass mit der Teilnahme und Rücksendung des Fragebogens der Erhebung und Verarbeitung der Daten gemäß dem beigefügten Informationsblatt zugestimmt wird.

## Ergebnisse

An der Umfrage haben 87 Personen teilgenommen, davon haben 62 den Fragebogen digital und 25 handschriftlich beantwortet. Eine Beantwortung musste auf Grund zu vieler fehlender Angaben aus der Auswertung ausgeschlossen werden. In den Fällen, in denen nur einzelne Antworten fehlten, wurden nur die betreffenden Fragen aus der Auswertung ausgeschlossen.

Es waren 36 der teilnehmenden Personen männlich (41,86 %) und 50 weiblich (58,14 %). Die CI-Tragenden waren im Alter von elf bis 88 Jahren (MW = 62,97 Jahre; SD = 15,75 Jahre). Regelmäßig ein Smartphone zu nutzen, gaben 71 Personen (82,56 %) an, die anderen 15 (17,44 %) verneinten diese Frage. Einen Anfahrtsweg zur betreuenden Klinik von unter 20 km gaben 39 Personen (45,35 %) an, 23 Personen (26,74 %) von 20 bis zu 40 km, 16 Personen (18,6 %) von 40 bis zu 60 km und acht Personen (9,3 %) von über 60 km. Es waren 62 der Befragten (72,09 %) unilateral implantiert und 24 der Personen (27,91 %) bilateral. Die Jahre der Implantation reichen von 1984 bis 2024, wobei 69 Personen (80,23 %) mindestens ein Jahr lang implantiert waren und sich somit in der Nachsorge befanden, während die anderen 17 Personen (19,77 %) noch in der Folgetherapie waren. Schon mal etwas von RC in der CI-Nachsorge gehört zu haben, gaben 15 Personen (17,44 %) an. Die anderen 71 Personen (82,56 %) hatten vor der Umfrage noch nichts davon gehört.

**Abb. 1:**
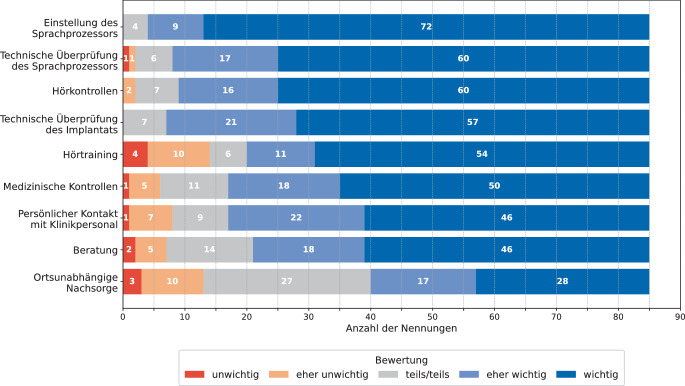
Relevanz verschiedener Aspekte in der CI-Nachsorge aus Sicht von CI-Tragenden (Frage 6, *n* = 85)

Das Ergebnis der *6.* Frage *„Wie wichtig sind Ihnen die folgenden Aspekte im Rahmen der Cochlea-Implantat-Nachsorge?“* ist in Abb. 1 dargestellt. Die Einstellung des Sprachprozessors haben fast alle Personen als wichtig erachtet, gefolgt von der technischen Überprüfung des Sprachprozessors, Hörkontrollen, der technischen Überprüfung des Implantats, Hörtraining, medizinischen Kontrollen, persönlichem Kontakt mit Klinikpersonal und Beratung. Die ortsunabhängige Nachsorge wurde im Vergleich zu den anderen Aspekten im Median nur als eher wichtig erachtet. Unter der Kategorie *Sonstiges* wurden die Argumente *Einfachheit, Social Media, Vorschläge und Anregungen, Material für das Hörtraining, Erreichbarkeit des Klinikpersonals* und *Ersatzteilversorgung* aufgeführt, welche als eher wichtig und wichtig eingestuft wurden.

**Abb. 2:**
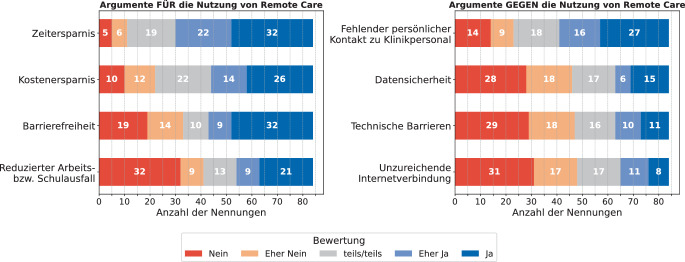
Argumente für und gegen die Nutzung von RC in der CI-Nachsorge aus Sicht von CI-Tragenden (Fragen 8 und 9, *n* = 84)

In Abb. 2 sind die Argumente für und gegen die Nutzung von RC in der CI-Nachsorge dargestellt (Frage 8: *„Sind die folgenden Aspekte aus Ihrer Sicht Argumente für die Nutzung von Remote Care in der Cochlea-Implantat-Nachsorge?“* und Frage 9: *„Sind die folgenden Aspekte aus Ihrer Sicht Argumente gegen die Nutzung von Remote Care in der Cochlea-Implantat-Nachsorge?“*). Während die Befragten Zeitersparnis im Median eher als ein Argument für die Nutzung von RC eingestuft haben, lag der Median bei den Argumenten Kostenersparnis, Barrierefreiheit und reduzierter Arbeits- bzw. Schulausfall bei teils/teils. Unter *Sonstiges* wurde als Argument für RC *Mobilität, ortsunabhängige Unterstützung, Termine auch bei Krankheit, Erlernen neuer Technologien, Verbesserung der Intensität des gezielten, individuellen Hörtrainings* und *Wegfall der Fahrten* genannt. Fehlender persönlicher Kontakt zu Klinikpersonal vor Ort wurde im Median eher als Argument gegen die Nutzung von RC eingestuft, während Datensicherheit, technische Barrieren und eine unzureichende Internetverbindung im Median eher nicht als Argument dagegen gesehen wurden. Unter *Sonstiges* wurden als Argumente gegen RC eine *mögliche schwierige Handhabung, Problematik für nicht technik-affine Menschen* und *Kompliziertheit* genannt.

**Abb. 3:**
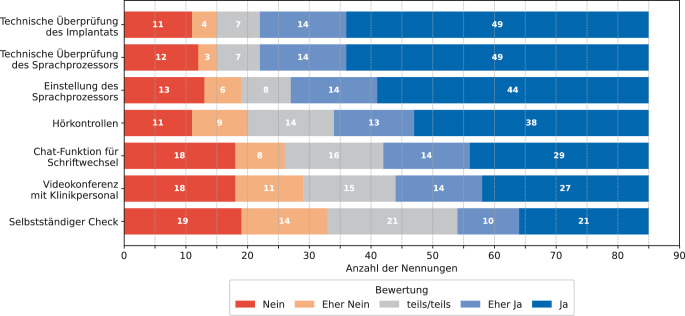
Interesse an RC-Funktionen in der CI-Nachsorge aus Sicht von CI-Tragenden (Frage 10, *n* = 85)

In Abb. 3 ist dargestellt, welche RC-Funktionen die Personen als interessant erachteten (Frage 10: *„Welche der folgenden Remote Care Funktonen in der Cochlea-Implantat-Nachsorge würden Sie gerne nutzen?“*). Dabei fällt auf, dass die meisten Personen Interesse an der Überprüfung des Implantats und der Überprüfung sowie Einstellung des Sprachprozessors in Form einer RC-Funktion haben. Hörkontrollen und eine Chat-Funktion für einen Schriftwechsel wurden im Median als eher interessant erachtet, während eine Videokonferenz mit dem Klinikpersonal und ein selbstständiger Check im Median als teils interessant und teils nicht interessant eingestuft wurden.

Unter *Sonstiges* wurden die Aspekte *Information über Neuheiten* und *Anleitungen zum Benutzen von Zubehör* genannt.

**Abb. 4:**
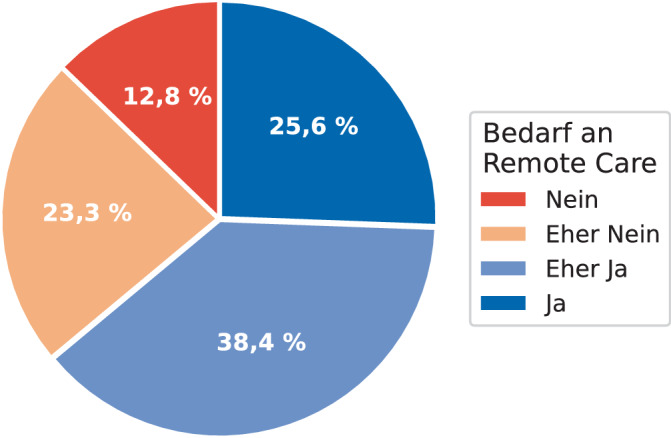
*„Würden Sie Remote Care im Rahmen der Cochlea-Implantat-Nachsorge in Anspruch nehmen?“* (Frage 11, *n* = 86)

Die abschließende Frage, ob die Personen RC in der CI-Nachsorge in Anspruch nehmen würden, haben 23 Personen (26,74 %) mit *Ja*, 33 Personen (38,37 %) mit *Eher Ja*, 20 Personen (23,26 %) mit *Eher Nein* und 11 Personen (12,79 %) mit *Nein* beantwortet (Abb. 4).

**Abb. 5:**
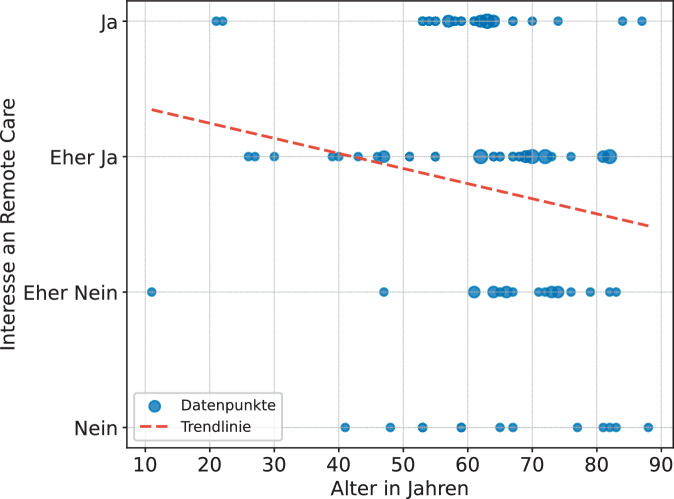
Signifikante Korrelation zwischen Alter und Interesse an RC (*p* < 0,05; *n* = 86)

**Abb. 6:**
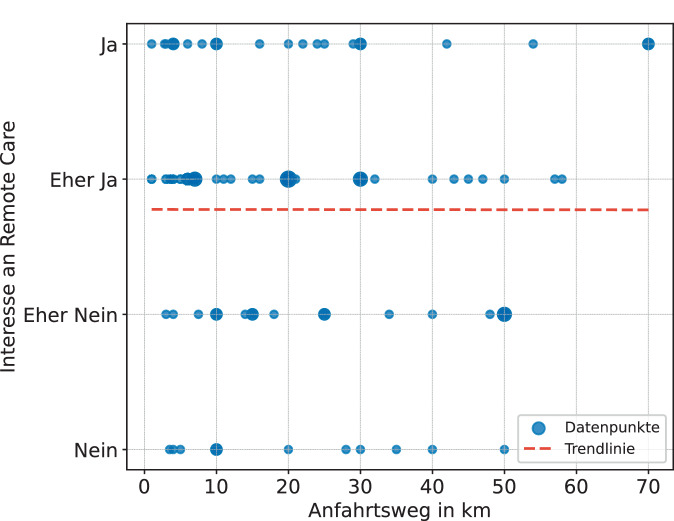
Keine Korrelation zwischen Anfahrtsweg und Interesse an RC (*n* = 80, exkl. sechs Ausreißer ≥ 90 km)

Die Ergebnisse wurden des Weiteren auf einen Zusammenhang zwischen Interesse an RC und den Aspekten Alter, Anfahrtsweg und CI-Erfahrung untersucht. Es konnte ein signifikanter Zusammenhang (*α* = 0,05) zwischen dem Alter der Teilnehmenden und dem Interesse an RC festgestellt werden. Wie in Abb. 5 ersichtlich, nimmt das Interesse an RC mit zunehmendem Alter signifikant ab (Rangkorrelation nach Spearman: *r*_*s*_ = −0,243, *p* = 0,024, *n* = 86). Dabei handelt es sich nach Cohen (1992) allerdings nur um einen schwachen Effekt. Zwischen dem Anfahrtsweg und Interesse an RC zeigt sich kein Zusammenhang (Rangkorrelation nach Spearman: *r*_*s*_ = −0,048, *p* = 0,674, *n* = 80), wie in Abb. 6 ersichtlich. Auch die Überprüfung auf den Zusammenhang von CI-Erfahrung (Jahr der ersten Implantation) und Interesse an RC ergab keinen Zusammenhang (Rangkorrelation nach Spearman: *r*_*s*_ = 0,052, *p* = 0,633, *n* = 86).

## Diskussion

Die Umfrage ergab, dass die Mehrheit der Befragten (64 %) RC in der CI-Nachsorge in Anspruch nehmen würde. Zudem nutzt ein Großteil der befragten Personen (83 %) regelmäßig ein Smartphone und erfüllt somit die Grundvoraussetzung für RC. Von den 15 Personen ohne regelmäßige Smartphone-Nutzung gaben elf an, RC nicht oder eher nicht nutzen zu wollen, während vier es dennoch in Betracht ziehen würden. Mit 64 % Zustimmung zur RC-Nutzung stimmen die Ergebnisse weitgehend mit den 63 % aus der Studie von Cochlear Ltd. überein [5]. Bemerkenswert ist, dass 71 von 85 Personen vor der Umfrage noch nichts von RC in der CI-Nachsorge gehört hatten und dennoch ein deutliches Interesse gezeigt wird.

In Bezug auf die wichtigsten Aspekte in der CI-Nachsorge wurde die Einstellung des Sprachprozessors von den Befragten als zentral eingestuft, gefolgt von der technischen Überprüfung des Sprachprozessors, Hörkontrollen und der technischen Überprüfung des Implantats. Die ortsunabhängige Nachsorge wurde im Vergleich zu den anderen Aspekten als weniger wichtig bewertet. Hier gab es ebenfalls die häufigsten neutralen Antworten (*n* = 27), was daran liegen könnte, dass einige Personen den Aspekt nicht richtig einordnen konnten (Frage 6 in Abb. 1). Bei der Frage nach interessanten RC-Funktionen standen die technische Überprüfung des Implantats sowie die technische Überprüfung und Einstellung des Sprachprozessors im Vordergrund, ebenfalls gefolgt von den Hörkontrollen (Frage 10 in Abb. 3). Diese vier Aspekte scheinen für CI-Tragende besonders wichtig zu sein und sollten bei der Integration von RC in den Klinikalltag besondere Beachtung finden. Der selbstständige Check wurde insgesamt als am wenigsten interessant eingestuft. Jedoch lagen auch hier mit *n* = 21 Nennungen die größte Anzahl an neutralen Antworten vor, was daran liegen könnte, dass viele Personen keine konkrete Vorstellung dieses Aspekts hatten. Die Einstufung des Interesses kann sich nach der praktischen Nutzung der genannten Funktionen verändern.

Die Bewertung der Argumente für RC fiel sehr unterschiedlich aus. Zeitersparnis wurde am häufigsten als Faktor für die Nutzung von RC genannt, reduzierter Arbeits- bzw. Schulausfall am häufigsten als nicht relevanter Faktor für die Nutzung von RC (Frage 8 in Abb. 2). Gleichzeitig bleibt der persönliche Kontakt zu Klinikpersonal vor Ort weiterhin bedeutsam. Das Fehlen dieses Kontakts war in der Umfrage das häufigste Argument gegen die Nutzung von RC (Frage 9 in Abb. 2). Datensicherheit, technische Barrieren und eine unzureichende Internetverbindung wurden von der Mehrheit der Befragten nicht oder eher nicht als Argument gegen die Nutzung von RC eingestuft. Die Ergebnisse lassen darauf schließen, dass ein großes Interesse an RC besteht, jedoch Termine vor Ort mit persönlichem Kontakt weiterhin relevant sind und RC eher als Ergänzung zu Vor-Ort-Terminen mit persönlichem Kontakt gesehen wird. Für einen Beleg dieser Hypothese hätten die Antwortmöglichkeiten der Frage 11 (Abb. 4) etwas differenzierter aufgeführt und in Optionen wie z. B. „ausschließlich“, „jedes 2. Mal“, „gelegentlich“, „selten“ und „nie“ unterteilt werden können.

Entsprechend der Erwartung konnte bestätigt werden, dass das Interesse an RC mit zunehmendem Alter signifikant abnimmt (Abb. 5). Diese Erkenntnis geht mit anderen Studien einher, die belegen, dass jüngere Personen eine höhere Nutzung und Akzeptanz telemedizinischer Gesundheitsanwendungen zeigen als ältere Personen [19]. Dennoch nimmt auch die Smartphone-Nutzung in der älteren Generation zu und beträgt bei den über 70-Jährigen noch 68,2 % [22]. Ein Zusammenhang zwischen der Länge des Anfahrtswegs und dem Interesse an RC konnte nicht belegt werden (Abb. 6). Auch die Überprüfung auf den Zusammenhang von CI-Erfahrung und Interesse an RC ergab keine Korrelation. Hier sollte berücksichtigt werden, dass viele der Befragten ihr Implantat innerhalb der letzten vier Jahre ab 2021 erhalten haben (63 %). Es wäre in einer erneuten Untersuchung mit einer größeren Datenlage zu prüfen, ob der Bedarf an persönlichem Kontakt mit zunehmender CI-Erfahrung abnimmt.

Die Datenerhebung erfolgte über einen Fragebogen, der sowohl handschriftlich in Papierform als auch digital beantwortet werden konnte. Diese duale Erhebungsmethode wurde gewählt, um eine breite Zielgruppe einzubeziehen und technik-averse Personen nicht auszuschließen. Die digitale Version führte aufgrund ihrer Konfiguration zu vollständigeren Antworten, während die Papierfragebögen teils fehlende Angaben aufwiesen. Trotz eines Informationstextes und der Möglichkeit zur handschriftlichen Beantwortung bleibt unklar, ob alle Befragten RC umfassend verstanden haben, insbesondere ohne vorherige Berührungspunkte zu dem Thema. Zudem könnte die Teilnahmebereitschaft durch die persönliche Einstellung zu RC beeinflusst worden sein. Desinteressierte Personen könnten die Umfrage eher ignoriert haben, während Befürworter motivierter waren, den Fragebogen auszufüllen. Dies könnte eine Verzerrung zugunsten positiver Einstellungen bewirkt haben. Außerdem ist anzumerken, dass kein standardisierter Fragebogen verwendet wurde, da in dieser Form, nach Kenntnis der Autorinnen und Autoren, keiner existiert. Da nur ein Kind (11 Jahre) mit Unterstützung eines Elternteils geantwortet hat, lassen sich keine Rückschlüsse auf den RC-Bedarf von Kindern oder deren Eltern ziehen. Diese methodischen Limitationen sollten bei der Interpretation der Ergebnisse und ihrer Generalisierbarkeit berücksichtigt werden.

Bislang existieren keine bekannten Rahmenverträge, die eine gezielte Kostenübernahme von RC in der CI-Nachsorge regeln. Die Kostenübernahme durch gesetzliche Krankenkassen ist bislang einzelfallabhängig und erfolgt nicht routinemäßig. Das Weißbuch CI-Versorgung [17] und die S2k-Leitlinie der DGHNO-KHC [6] empfehlen eine verstärkte Qualitätssicherung und standardisierte Prozesse, bisher jedoch ohne Regelungen zu RC und Fernanpassungen. Diese Arbeit zeigt, dass der Bedarf an RC in der CI-Nachsorge gegeben ist und damit einhergehend auch der Bedarf einer standardisierten Kostenübernahme durch die gesetzlichen Krankenkassen vorliegt.

## Ausblick

Fast zwei Drittel der Befragten befürworten die Nutzung von RC, betonen jedoch zugleich die Bedeutung des persönlichen Kontakts zum Fachpersonal. Dies verdeutlicht die Notwendigkeit einer sorgfältigen Implementierung, um RC als Ergänzung zu Vor-Ort-Terminen in der CI-Nachsorge zu etablieren. Mit der zunehmenden Smartphone-Nutzung in allen Altersgruppen wird die routinemäßige Einbindung von RC in die CI-Nachsorge künftig eine zentrale Rolle einnehmen. Die Kombination verschiedener RC-Modelle kann die Nachsorge effizienter gestalten und gleichzeitig Ressourcen in Kliniken und Rehabilitationszentren schonen. Obwohl RC den persönlichen Kontakt nicht ersetzen kann, bietet es als Ergänzung eine innovative Lösung, die den wachsenden Anforderungen der digitalen Gesundheitsversorgung gerecht wird und zukünftig immer mehr Nachfrage finden wird.

## Fazit für die Praxis

- Die Ergebnisse dieser Untersuchung verdeutlichen ein Interesse an Remote Care (RC) in der Cochleaimplantat(CI)-Nachsorge, und ein Großteil der CI-Tragenden erfüllt die grundlegende Voraussetzung für die Anwendung dieses Modells.
- Das stärkste Argument für die Nutzung von RC ist Zeitersparnis für CI-Tragende.
- Der persönliche Kontakt zu Fachpersonal in Kliniken und Rehabilitationszentren wird von CI-Tragenden weiterhin als wichtig erachtet.
- Der Bedarf an RC nimmt mit zunehmendem Alter ab, und besonders die älteren CI-Tragenden bevorzugen eine Nachsorge vor Ort.
- Insgesamt ist RC ein zukunftsorientiertes Modell, und die Implementierung von RC als Ergänzung zu Vor-Ort-Terminen in den Klinik- und Rehabilitationsalltag findet Nachfrage bei CI-Tragenden.

## Data Availability

Die erhobenen Datensätze sind in anonymisierter Form bei der korrespondierenden Autorin auf Anfrage verfügbar.
